# The Association Between High Body Mass Index and Technology Use Among Female Elementary School Students

**DOI:** 10.7759/cureus.11903

**Published:** 2020-12-04

**Authors:** Mesbah Jari Alshumrani, Amal Yousef Alhazmi, Samar A Baloush, Shahad O Aljohani, Wejdan T Almutairi

**Affiliations:** 1 College of Medicine, King Saud Bin Abdulaziz University for Health Sciences, Jeddah, SAU

**Keywords:** body mass index: bmi, pediatrics, obesity, screen time, socioeconomic, physical activity, females, elementary school

## Abstract

Background

Overweight and obesity among children are progressively turning into global issues. Numerous factors have been linked to the proliferation of pediatric obesity. However, there are still questions as to whether the corresponding proliferation in the use of technology could be linked to the increase in body mass index (BMI) among children. The aim of this study is to investigate the association between high BMI in female elementary school-age children and time spent using electronic devices.

Methods

This is a cross-sectional study that was conducted across three female elementary schools located in Jeddah between 2017 and 2018. All schools which are affiliated with the Ministry of National Guard in Jeddah were included. Demographic data, school performance, sleep routine, physical activity, parents' beliefs, and family demographic data were collected from the legal custodian of the children. The research group visited schools to obtain children's BMI measurements.

Results

The number of participants who responded to the survey was 681. The results showed that the increase in BMI was non-significantly linked with the period spent using electronic devices, the use of electronic devices before sleep, nor the kind of device used. However, there was a significant association between high BMI and one type of electronic device: the television. There was a significant association between high BMI and the denial by parents that their children were obese. Furthermore, 50% of children were obese while 92.9% of overweight children's parents did not believe that there was a problem with the weight of their children.

Conclusion

There is no significant association between screen time spent on electronic devices and high BMI among female elementary school-age children. However, lack of physical activity, fast food consumption, and genetic predispositions are still major contributing factors for childhood obesity and overweight. Nearly all parents of children who are overweight did not perceive their children as being overweight, which brings to the fore the subject of lack of awareness about childhood obesity among parents.

## Introduction

Childhood obesity is considered a universal epidemic [1]. By 2016, statistics by the World Health Organization were showing that the number of obese or overweight children was around 41 million while another 92 million children faced the risk of becoming overweight [2]. The same source indicates that childhood obesity and overweight prevalence had increased significantly to 6.7% from 4.2% in the last two decades. Other estimates indicate that children who are at the risk of high body mass index (BMI) are around 60 million [2,3]. With regards to Saudi Arabia, the prevalence of obesity and overweight in children between the ages of five and 18 was 23% and 9.3% in 2010, respectively [4]. BMI is a method used in determining the healthy weight of individuals. The calculation of BMI is accomplished by dividing the weight in kilograms by the square of height in meters. Using the results of these calculations, children are placed into numerous categories: obese, overweight, normal, and underweight [5]. A BMI for age equivalent to or above the 85th percentile but below the 95th percentile is recorded as overweight. Furthermore, when the BMI is equivalent to or above the 95th percentile, the child is said to be obese [5].

The Oxford Dictionary defines screen time as "time spent using a device such as a computer, television, or games console" [6]. For children between the ages of five and 14 years, recommended screen time is between one and two hours a day [7]. However, in recent times, the amount of physical activity that children are involved in has been affected by technological developments and may predispose to childhood obesity [8]. There are several studies that have been done in different countries that attempt to elucidate on the link between screen time and high BMI. One such study was done in 2015 in Canada, and its results show that when screen time exceeds the daily recommended limits, it is accompanied by a poor choice of food [9]. In Saudi Arabia, a study conducted at the school health clinic in King Abdulaziz Housing for National Guard in Riyadh showed that children that spend over 180 minutes watching television per day on the weekends tend to have higher BMI. It also shows that there was an escalation in the risk of obesity among children that watch television for more than 180 minutes daily [10].

Obesity and overweight in childhood could result in obesity in adulthood as two-thirds of children with a high BMI can grow up to be overweight or obese adults [11]. As a result, adults who are overweight have been shown to have increased risk of being affected by psychological disorders, cardiovascular diseases, diabetes, and musculoskeletal diseases [12]. Currently, technology has turned into an essential element of life. The increased demand in the use of technology could have an impact on BMI. It is on this basis that this study seeks to determine the link between technology use and children's BMI. This study will also look at other factors that have an impact on BMI such as diet, sleep, physical activity, and socio-economic status. The study also focuses on increased BMI and socio-economic factors, which seems to have been generally overlooked in previous studies. Also, it is noted that obesity constitutes a risk factor for many diseases that include cardiovascular conditions which can be preventable [13]. Thus, this study will assist providers of healthcare services to put in place a suitable management plan based on recent information.

## Materials and methods

Study population and design

This is a cross-sectional study that involved three female elementary schools in Jeddah, Saudi Arabia. All the included schools are affiliated with the Ministry of National Guard in Jeddah childhood clinic. The data collected was from 2017 until 2018. The study was approved by the King Abdullah International Medical Research Center (KAIMRC) ethical committee. The students' school grades which were included in the study were from grade one to grade six. Meanwhile, we excluded students that did not provide any custodian consent as well as incomplete data. The calculation of this study's sample size used a population of 650,560. This is the number of Jeddah's female school children which was provided to us by the General Authority for Statistics over a phone call inquiry from their headquarters. The study’s confidence level is 95%, and the margin of error is 5%. The estimated size of the sample was n=384, with the actual sample size being 681.

Demographic variables and survey

Data was distributed based on several elements linked to the lifestyle of the children on diet, performance at school, physical activity, diseases, use of technology, sleep patterns, and diseases. In addition, demographic information about parents including age, income, and level of education were recorded. The collection of information was done through a self-administered survey that was completed by one of the parents or a legal guardian. To obtain information about diet, the respondents had to give information about the frequency of daily meals, intake of vegetables and fruits, fast food, sugary and/or soft drinks, and fresh juices. Meanwhile, physical activity data were gathered by asking about the type, location, and duration of physical activity. Assessment of sleep patterns was accomplished by determining the quantity of sleep hours in a day, frequency of naps, and fragmented sleep. Data regarding the use of technology was gathered through enquiring about the number of hours that the children spent using technology and the kind of devices used. The survey validation was assessed by the members of the KAIMRC committee. Lastly, BMI measurements were done by the research team by conducting school visits. It was measured by taking the weight and height of the students using a weight scale and a stadiometer. To get the BMI, weight in kilograms was divided by height in meters squared and was charted on the Centers for Disease Control and Prevention (CDC) universal chart.

Statistical analysis

We summarized the data using Statistical Package for Social Sciences (SPSS) version 26 (IBM Corp., Armonk, NY, USA) and simple descriptive statistics were reported by Microsoft Excel 2018. Regarding bivariate analysis, the four BMI categories were represented by the variable qualitative: underweight, normal, overweight, and obese. Regarding the other variable, it was screen time hours spent by the children. Percentages and frequencies were used for qualitative data. The relationship between different variability was investigated using chi-square test, correlation analysis, and Fisher's exact test.

Ethical considerations and data collection

In this study, participation was voluntary as each family received a notification letter which contained information regarding the study team, objectives, and requesting consent to measure the children's weight and height. The legal guardians also received an Arabic version of the survey to decide whether to participate or not in the research. After data collection, the research team translated the data that was collected in Arabic into English. The study team also assured the anonymity and confidentiality of the participants. No personal data, like names and contact numbers of the participants, was collected. Each child was given a serial number, which was on the cover of the data collection and survey file. From the children's side, verbal consent was obtained to take their measurements beforehand. Lastly, all data was stored in one workplace computer to ensure confidentiality and privacy. Accessing the information on the computer was only permitted for the research team and was later stored in the research office. The research team presented the data honestly, ensuring that the study was impartial and independent.

## Results

**Figure 1:**
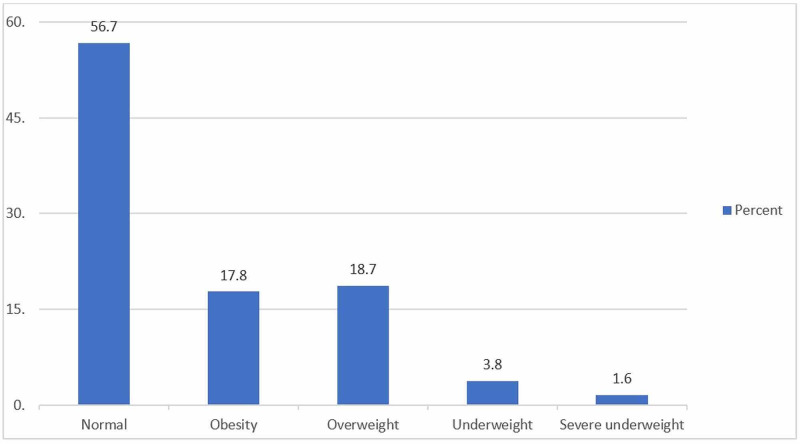
BMI categories among female elementary school students

**Figure 2:**
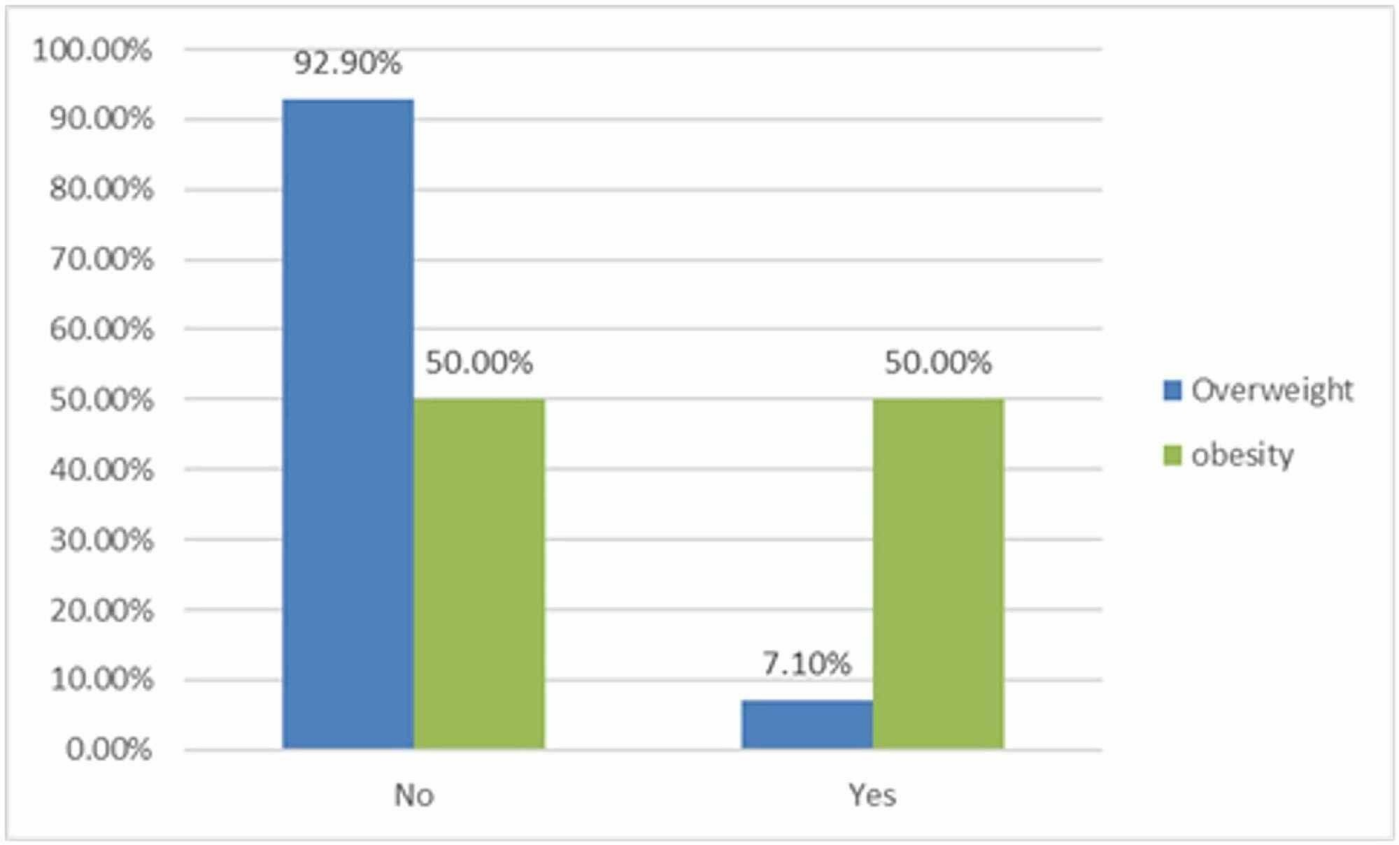
Parental beliefs regarding children’s obesity

A total of 681 school girls were included in the study between grades one to six. Only complete surveys were included in the study. The BMI for nearly half (56.7%) of the study population was normal. Overweight, obesity, underweight, and severe underweight were found to be 18.7%, 17.8%, 3.8%, and 1.6%, respectively (Figure 1). Parents were also requested to indicate if they perceived their children as overweight. Among the parents of overweight children, 92.9% said that they did not believe their child was overweight. Among the parents of obese children, only 50% indicated that they believe that their child was obese (Figure 2).

**Figure 3:**
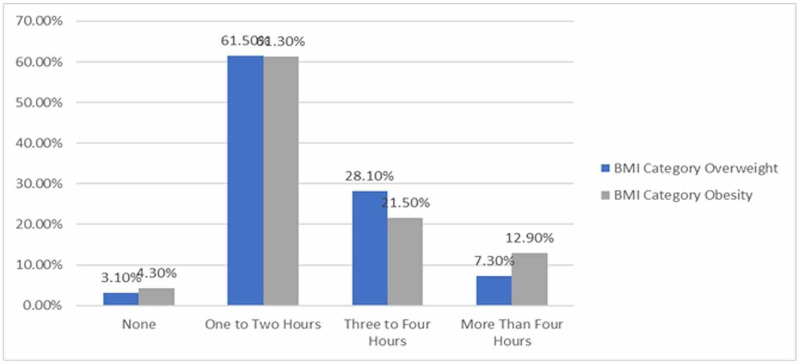
Daily exposure to electronic devices duration among obese and overweight children.

Among the children, 63.6% of them indicated that they had permission to use electronic devices from one to two hours a day while the proportion of participants that did not use electronic devices was 2.3%. Meanwhile, the percentage of children using devices for over three hours a day was 25.8%. Only 8.3% indicated that they used devices for more than four hours a day. With regards to the obese and overweight participants and the time they spent on electronic devices, over half of the obese and overweight children had between one and two hours of screen time a day (Figure 3). Obese and overweight children who used electronic devices for three to four hours constituted of 28.1% and 21.5%, respectively. Based on the collected data, the proportion of children who used devices for less than one hour or never used them were 3.10% and 4.30%, respectively. Only 7.30% of obese females and 12.90% of overweight children used devices for more than four hours a day. The association between the type of device used, time spent using an electronic device, and the use of electronic devices before sleep was non-significant. However, there was a significant association between high BMI and one kind of device: the television (P-value = 0.044).

Diet and physical activity

There was a significant association between the number of fast-food meals in a week and an increased BMI (P-value = 0.017) (Table 1). Between other dietary factors like the quantity and quality of meals, eating in front of the television, consumption of vegetables and fruits, fresh juices, soft drinks, and canned juices, there was a non-significant association. Furthermore, results showed a significant association between obesity and a lack of physical activity (P-value = 0.025). In this case, 65.6% of obese children were not involved in any type of physical activity while 75.8% of obese children and 69.1% of overweight children did not participate in any kind of physical education classes at school (P-value = 0.031).

**Table 1:** The link between high BMI and use of electronic devices, obesity, physical activities, and diet. BMI: Body Mass Index, * Significant Value set of less than 0.05

Category			BMI Category		P-value
			Overweight	Obesity	
Diet	Fast food Meals	Do Not Eat	29.2%	18.5%	0.017*
		One class	28.7%	15.4%	
		Two classes	2.1%	6.6%	
		Three classes	0.0%	2.2%	
Electronic Use	Television	No	37.1%	51.6%	0.044*
		Yes	62.9%	48.4%	
Obesity	Parents Believe Their Child is Obese	No	92.9%	50.0%	0.001*
		Yes	7.1%	50.0%	
	Obese Member in The Family	No	80.6%	60.9%	0.005*
		Yes	19.4%	38.0%	

Diseases and comorbidities

With regards to associated diseases and obesity comorbidities, there was no significant association between obesity and overweight with the following conditions: asthma, epilepsy, diabetes mellitus, vitamin D deficiency, autism, recurrent upper respiratory tract infection, sleep apnea and bone fracture.

Family effects

Female students who have family members that are obese are at a substantially higher obesity risk (P-value = 0.005). The association between high BMI, the age, type of house, income, educational level of parents, and the child’s number of siblings was non-significant. Moreover, there was a significant association between high BMI and the denial by parents that their children were obese (P-value = 0.001) where 50% of obese students and 92.9% of overweight children's parents did not believe that there was a problem with their child's weight.

Sleep, school performance, and use of electronics

The association between BMI ≥ 25 kg/m^2^ and daily sleep hours, fragmented sleep, taking naps and length of naps, and snoaring was non-significant. In addition, there was no significant association between BMI ≥ 25 kg/m^2^ and performance at school, challenges with speech, bullying, and social difficulties. Lastly, watching television was very common between obese and overweight children. 62.9% of obese females and 48.8% of overweight female students showed significant association with television watching (P-Value = 0.044) (Table 1).

## Discussion

Aa obesity now considered a global health challenge, it inflicts a substantial risk with regards to the health of future generations as a result of the link between obesity and cardiac, endocrine, and other medical challenges [8,12]. Notwithstanding efforts by the government to lessen the rate of obesity in the Middle East, the Gulf region is still home to the highest rates of obesity among children [12,14]. It is on this basis that a better comprehension of obesity risk factors in this area must be the initial step towards reducing the burden imposed by obesity from a young age to better combat this challenge.

In our study, it was revealed that there was no association between the use of electronic devices and the kind of device used in general. However, it has been noted that there is a significant link between high BMI and the time spent watching television. There are different settings that have been used to evaluate obesity risk factors. In 2018, Mazidi et al. researched the prevalence of obesity among childhood and adolescents by conducting a meta-analysis and a systematic review on studies done in Asia and indicated that obesity and overweight prevalence among girls between the ages of five and 11 years was 4.8% and 10.9%, respectively [15]. Another systematic review was conducted in Brazil and concluded that the obesity prevalence varied in different regions, with a range of 10.4% to 28.8% among children aged two to 19 years [16]. In Europe, a study on prevalence of obesity showed that 15.6% of school-aged children between six to 11 years old were obese while the prevalence rate of overweight was 4.9% [13]. Even though our study did not include male children, almost one-third of our population was either obese (18.7%) or overweight (17.8%), which is slightly higher than other compared countries as stated above. This is supported by studies that demonstrated how obesity is increasing in Saudi Arabia among children [4,10]. In Al-Khobar city, a study involving children between the ages of six and 17 years showed that overweight prevalence was 20% while that of obesity was 11% [17]. Another Saudi study done in various areas showed a 6.74% obesity and a 12.7% overweight prevalence in female children and adolescents [18]. In the city of Jeddah in Saudi Arabia, a cross-sectional study done among patients aged between two and 18 years indicated a 23.1% overweight prevalence and a 9.3% obesity rate [19].

With regards to obesity and the use of electronic devices, a study done by Hancox and Poulton concluded that a direct link exists between watching television and BMI in girls [20] and several studies demonstrated the same conclusion [10,21-23]. Such findings support the results of this study, which showed that there is a positive association between high BMI and watching television. Moreover, a study done in the western region of Saudi Arabia by Alagha showed that children spending more than 120 minutes daily watching television had a higher BMI than those watching television less than 120 minutes daily. However, the same study did not indicate any significant association between increased BMI and time spent watching television (P-value = 0.477). Furthermore, Alagha et al. concluded that there was an inverse proportion between time spent doing physical activity and BMI [24]. In opposition, the current study showed that there is a positive correlation between BMI categories and the level of physical activity.

Also, the present study evaluated numerous socio-economic elements such as the age of parents, their level of education, obesity of other family members, house type, and the child's number of siblings. Among these factors, the only factor which showed to be significantly associated with high BMI was the existence of obesity in the family. Fuemmeler et al. found that children with two obese parents are 10 to 12 times more likely to develop obesity [25]. This puts a great emphasis on the role of parents or family in choosing a healthy lifestyle since childhood to prevent developing obesity among their children.

The present study faces several limitations. For instance, the significance of the outcomes can be questioned since the study is cross-sectional. Also, the study was done in a single city, a situation that could make the external validity of the findings to be questioned. Furthermore, the term "watching television" can be very subjective from one family to another with how advanced technology is nowadays. School performance, grades, and bullying can also be seen differently from the parent's point of view. Lastly, the targeted population was only females, and including males might have given us more insight into the habits of both genders.

## Conclusions

The association between BMI and screen time spent on electronic devices is non-significant among female elementary school-age children. Although obesity and overweight are serious health issues that have long- and short-term effects, parents of children who are overweight did not perceive their children as being overweight, which shows lack of awareness among parents with regards to the bodies of their children. For such challenges, national programs are necessary to increase community awareness toward childhood obesity and its long-term consequences. Furthermore, weight management health services are an essential preventive measure to evaluate and monitor children with overweight and obesity, giving the increasing childhood obesity prevalence in young age groups.
